# “Green spectrophotometric determination of rupatadine, its toxic impurity, and methyl paraben: comparative AI-assisted univariate versus multivariate chemometrics”

**DOI:** 10.1038/s41598-026-68686-1

**Published:** 2026-09-09

**Authors:** Salma S. Mansour, Amr M. Mahmoud, Azza A. Moustafa, Nancy W. Nashat

**Affiliations:** Pharmaceutical Analytical Chemistry Department, Faculty of Pharmacy, Cairo University, Kasr El-Aini St, Cairo, 11562 Egypt

**Keywords:** Rupatadine fumarate, Dual wavelength, Artificial neural network, Partial least squares, Multivariate curve resolution, Green Analytical chemistry, GLANCE, Biochemistry, Chemistry

## Abstract

Sensitive and selective green UV-spectrophotometric univariate and multivariate chemometric methods were developed to resolve the overlapped spectra of rupatadine fumarate (RUP), its impurity desloratadine (DES), and methyl paraben (MP). The univariate approach employed was the dual-wavelength (DW) method. Guided by artificial intelligence (AI) in the selection of the optimal wavelength pair, the determination of RUP was performed at 230 and 284.3 nm, and for MP at 236.6 and 253.8 nm. Among the multivariate methods, ANN demonstrated superior predictive accuracy (lowest RMSEP values), followed by PLS-1 and MCR-ALS, highlighting the power of machine learning for complex mixture resolution, particularly in the presence of the preservative MP. A three-factor, five-level experimental design was adopted to construct a calibration set comprising 25 mixtures with varying component ratios, along with 6 independent validation mixtures. The developed methods were successfully applied to pharmaceutical formulation containing the studied drug and were validated in accordance with International Conference of Harmonization (ICH) guidelines. The results obtained were reliable and reproducible, confirming the suitability of these methods for routine analysis and quality control in laboratories. Moreover, the GLANCE tool (Graphical Layout for Analytical Chemistry Evaluation) offered a clear visual summary of the twelve structured method attributes.

**Supplementary Information:**

The online version contains supplementary material available at https://doi.org/10.1038/s41598-026-68686-1.

## Introduction

**Fig. 1:**
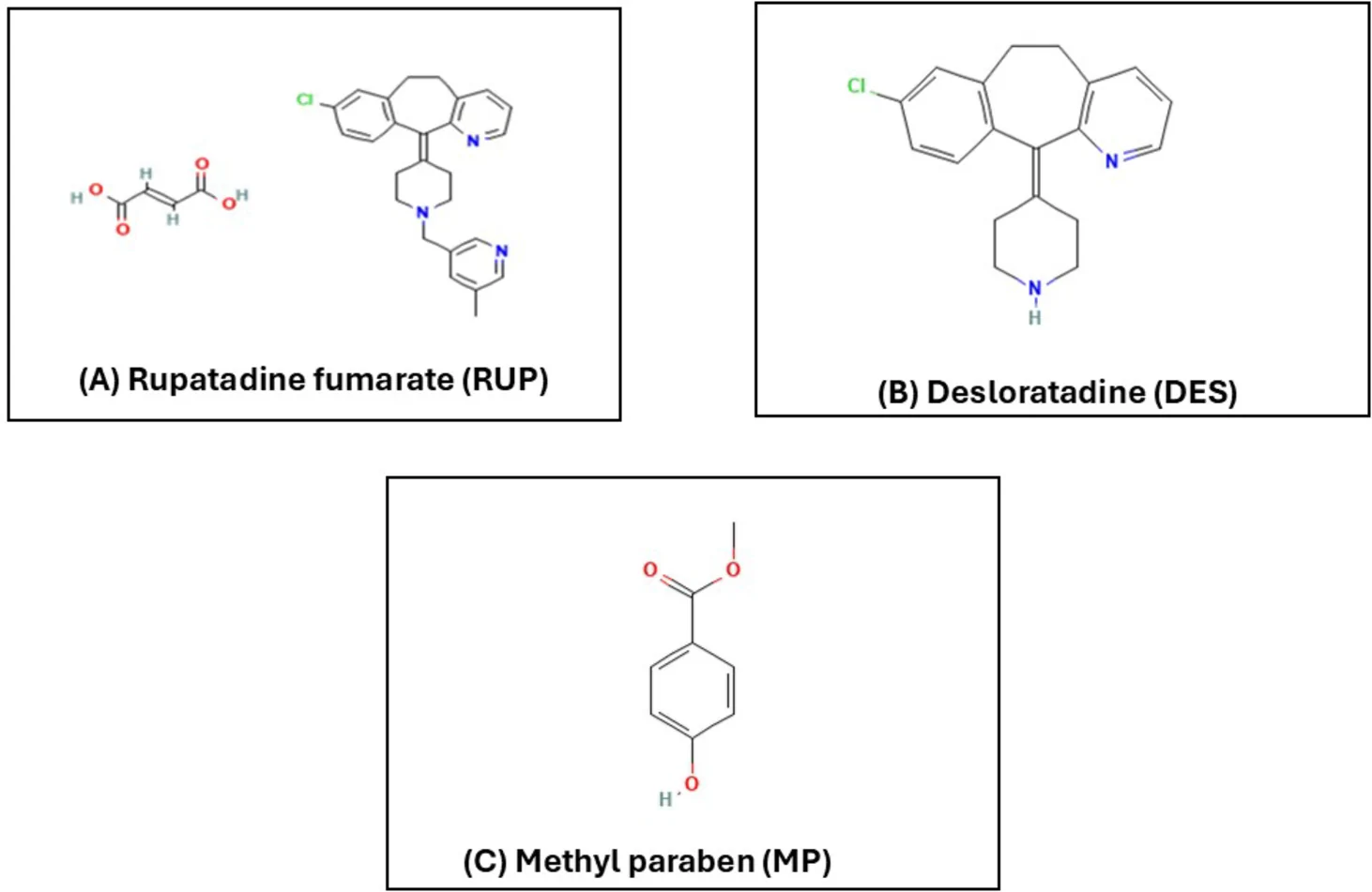
Chemical structure of Rupatadine fumarate (RUP), Desloratadine (DES), and Methyl paraben (MP).

The presence of undesirable or potentially harmful analytes in pharmaceutical products is a major concern. This includes specific impurities, which are subject to strict regulatory control due to direct health hazards, and certain preservatives linked to adverse effects^1,2,3^. Therefore, there is an urgent need for simple, rapid, and reliable methods for the assay of these compounds^4^. Rupatadine fumarate, 8-Chloro-11-[1-[(5-methylpyridin-3-yl)methyl]piperidin-4-ylidine]-6,11-dihydro-5 H-benzo[5,6]cyclohepta[1,2-b]pyridine(2E)-but-2-enedioate (RUP, Fig. 1A**)** is a second-generation antihistamine with platelet-activating factor (PAF) antagonist activity^5^. It is commonly used for the treatment of chronic urticaria, allergic rhinitis^6^, and conjunctivitis^7^. RUP is an effective option for treating seasonal allergic rhinitis, as it improves both nasal and non-nasal symptoms^8^. Desloratadine, 8-chloro-6,11-dihydro-11-(4-piperdinylidene)- 5* H*-benzo[5,6] cyclohepta[1,2-b]pyridine (DES, Fig. 1B), is identified as impurity B of RUP in the British Pharmacopeia (BP)^9^ and has also been reported as a degradation product of the drug^10^. The BP has established a maximum permissible concentration of 0.5. Methylparaben (methyl 4 hydroxybenzoate, MP) is a frequently employed preservative in rupatadine formulations (Fig. 1C**)**. Parabens, including MP, are widely used as preservatives and antimicrobial agents^11^ but have been linked to health concerns such as endocrine disruption, breast cancer, and reproductive issues^12,13^. Some studies report that these compounds may be carcinogenic due to their estrogenic and antiandrogenic activities^12,14^. Consequently, regulatory guidelines now restrict the use of parabens, obliging manufacturers to justify their necessity and monitor their safety.

Various chromatographic, spectroscopic, and electrochemical techniques have been reported in literature for the determination of RUP [14–16] and DES [16–18]. Additionally, simultaneous determination of RUP and DES has been reported using several methods including chromatography and spectroscopy^15^. To date, only one micellar High performance liquid chromatography (HPLC) method has been reported for the simultaneous determination of RUP, DES, and MP^16^.

Spectrophotometric methods are widely used in pharmaceutical analysis because of their simplicity, rapidity, cost-effectiveness, and suitability for routine quality control^17,18^. However, their application is often limited by spectral overlap between drugs, impurities, preservatives, or excipients, particularly when structurally related compounds are present. To overcome this challenge, numerous univariate spectrophotometric and multivariate chemometric methods have been developed for the analysis of complex overlapping mixtures^13,19–24^.

The dual-wavelength method is one of the simplest univariate spectrophotometric approaches for resolving overlapping spectra, where the absorbance difference at two selected wavelengths is proportional to the concentration of one analyte while eliminating interference from another^25–27^. Alternatively, multivariate chemometric techniques enhance the resolution of highly overlapping spectra by extracting meaningful information from spectral data^20,28^. Among the most widely used algorithms are partial least squares (PLS), artificial neural networks (ANN), and multivariate curve resolution–alternating least squares (MCR-ALS). PLS-1 constructs latent variables that maximize the covariance between spectral data and analyte concentration, providing robust predictive performance for single-analyte calibration^24,29–31^.

ANN are machine learning models composed of input, hidden, and output layers. The absorbance inputs and corresponding predicted concentrations are represented by interconnected nodes, referred to as neurons, with weights optimized during training to predict concentrations. Once trained, the fixed network structure is used for subsequent predictions^32–34^. MCR builds a regression model by relating each variable to others, effectively extending the Beer–Lambert law to the multi-wavelength domain. MCR decomposes the spectral data into concentration profiles and pure spectral matrices, followed by the calculation of the residual matrix^35^. The ALS algorithm is specifically designed for the iterative resolution of bilinear models by alternately solving for concentration and spectral profiles in a constrained optimization framework. Since matrix decomposition is inherently non-unique, constraints such as unimodality, closure, equality, or non-negativity are applied to restrict the solution space and yield chemically interpretable profiles^23,36^.

Green Analytical Chemistry (GAC) principles were considered throughout this study^37^. Several metrics have been developed to assess the greenness of analytical methods, including the Analytical Eco-Scale (AES), GAPI, MoGAPI, Complex MoGAPI, and AGREE, each with specific advantages and limitations. In this work, the Analytical Green Star Area (AGSA) was selected because it combines visual assessment with quantitative scoring while maintaining full alignment with the 12 principles of GAC, providing a comprehensive and objective evaluation of method greenness^38^.

AI-assisted optimization of the univariate method^39^,alongside chemometric techniques, reduced the experimental burden. This resulted in decreased reagent use, waste production, and energy and time demands.

To provide a clear and concise overview of our developed method, we employed the GLANCE (Graphical Layout Tool for Analytical Chemistry Evaluation) framework^40^. This visual tool uses a standardized template to summarize an analytical method across twelve essential dimensions: novelty, analytes, sample preparation, reagents, instrumentation, method validation, matrix effects and recoveries, real sample applications, analytical metrics, main results, limitations, and additional information. GLANCE improves clarity, facilitates method comparison, and enhances scientific communication by integrating key method features into a single, editable visual format.

This study aimed to develop the first green spectrophotometric methods, including an AI-assisted univariate approach and three multivariate chemometric models (PLS-1, ANN, and MCR-ALS), for the simultaneous determination of RUP, DES, and MP. The methods were compared in terms of their ability to resolve highly overlapping spectra, with particular emphasis on the sensitive and selective determination of DES and MP. In addition, the greenness of the proposed methods was evaluated using the AGSA metric, and the GLANCE framework was employed to provide a comprehensive assessment of their analytical performance and sustainability.

## Materials and methods

### Instrument and software

The absorbance measurements were carried out using a SHIMADZU dual-beam UV–visible spectrophotometer (model UV-1650, Kyoto, Japan) equipped with 1 cm quartz cells. Spectral data were automatically recorded through the UV-Probe software (version 2.21, SHIMADZU). Univariate method optimization was facilitated using Microsoft Copilot (“https://copilot.microsoft.com”), an AI-powered computational assistant^41^. Multivariate data analysis was performed using MATLAB^®^ 9.2.0.538062 (R2017a). Partial Least Squares (PLS) analysis was performed using the PLS Toolbox 2.1 (Eigenvector Research Inc., Manson, WA, USA), while Artificial Neural Network (ANN) modeling utilized the MATLAB Neural Network Toolbox. The MCR-ALS graphical user interface was accessed online at http://www.mcrals.info.

### Chemicals and solvents

RUP was graciously supplied by ATCO Pharma, Cairo, Egypt. Its purity was assessed and found to be 100.4 ± 0.73%^42^. DES was supplied by Global NAPI Pharmaceuticals, and its purity was determined and found to be 100.08 ± 0.643%^43^. MP was obtained from GlaxoSmithKline, and its purity was found to be 98.01 ± 0.1%^44^. Ethanol of HPLC grade (Fisher Chemical, United Kingdom) was utilized as the solvent for all spectrophotometric measurements. Alergoliber syrup was manufactured by ATCO Pharma and was labeled to contain 1.28 mg mL ^-1^ Rupatadine fumarate(equivalent to 1 mg mL^-1^ Rupatadine ).

### Stock solutions

Standard solutions of RUP, DES, and MP (0.5 mg mL^− 1^) were prepared by accurately weighing 25 mg of each compound into separate 50 mL volumetric flasks and completing the volume with ethanol.

### Procedures

#### Spectral characteristics of RUP, DES, and MP

Zero-order (D₀) absorption spectra of RUP, DES, and MP (10 µg mL⁻¹ each in ethanol) were recorded. Ethanol served as a blank over the wavelength range of 200–400 nm.

#### Dual wavelength (DW) method

Aliquots corresponding to 40–400 µg of RUP and 30–200 µg of MP were individually taken from their respective standard working solutions (0.5 mg mL ^-1^ and transferred into two separate sets of 10 mL volumetric flasks. Each flask was then diluted to volume with ethanol to yield final concentration ranges of 4–40 µg mL⁻¹ for RUP, and 3–20 µg mL⁻¹ for MP. The zero-order spectra of each were then recorded.

The differences in amplitudes of the resulting spectra were measured at 230 and 284.3 nm for RUP (ΔP = A₂₃₀ – A₂₈₄.₃) and at 236.6 and 253.8 nm for MP (ΔP = A_₂₃₆.₆ –_ A_₂₅₃.₈)_. Calibration curves were constructed by plotting the measured amplitude differences at the selected wavelength pairs against the corresponding concentrations of RUP and MP, respectively, and the associated regression equations were calculated. Different laboratory-prepared mixtures containing varying ratios of RUP and MP were analyzed. The procedures previously outlined for the DW methods were applied. The concentrations of RUP and MP in the prepared samples were then determined using the corresponding regression equations.

#### Construction of calibration and validation sets for chemometric methods

A three-factor, five-level experimental design was employed to construct the calibration model. The calibration set consisted of 25 mixtures containing various combinations of high and low concentrations of the studied drug, impurity, and preservative, along with an independent validation set of six mixtures^45^. The design levels were coded from − 2 to + 2, with center values of 2 ,4 ,5 ,6 ,8 ,10 µg mL⁻¹ RUP, and MP, while 1 ,2 ,3 ,4 ,5 µg mL⁻¹ for DES, respectively, as shown in Table 1. Different aliquots of the previously prepared standard solutions were transferred into 10 mL volumetric flasks, and the volume was adjusted to the mark with ethanol to obtain the required mixtures. UV spectral data were collected over the range of 200–400 nm, with the 300–400 nm region excluded from analysis. The selected data were then exported to MATLAB for processing. Calibration models were constructed using PLS-1, ANN, and MCR-ALS techniques. An additional set of six mixtures was used to evaluate and compare the predictive performance of the developed models.

**Table 1:** Concentrations of RUP, DES, and MP in the prepared mixtures (µg mL^− 1^).

Mixture No.	RUP	DES	MP
1	6	3	6
2	6	1	4
3	2	2	2
4	4	1	10
5	5	5	10
6	10	5	6
7	10	3	4
8	6	2	10
9	4	5	4
10	10	2	8
11	4	4	8
12	8	4	6
13	8	3	10
14	6	5	8
15	10	4	10
16	8	5	2
17	10	1	2
18	2	1	6
19	2	3	8
20	6	4	2
21	8	1	8
22	2	4	4
23	8	2	4
24	4	2	6
25	4	3	2
26	2	2	4
27	3	2	0.5
28	6	6	6
29	5	2	5
30	6	3	6
31	8	4	8

* Shaded columns are the validation set mixtures.

#### Application of the proposed methods for determination of RUP in Alergoliber^®^ Syrup

An aliquot of 3.91 mL of Alergoliber^®^ Syrup, containing 5.00 mg RUP, was transferred into a 50 mL volumetric flask. The volume was completed to the mark with ethanol and sonicated for 20 min to ensure complete dissolution. The solution was then filtered to have a stock of 0.1 mg mL^− 1^. Subsequent serial dilutions were prepared to fall within the established linearity ranges, and these diluted samples were analyzed using the proposed methods. The concentrations of RUP were then predicted according to the applied methods.

## Results and discussion

**Fig. 2:**
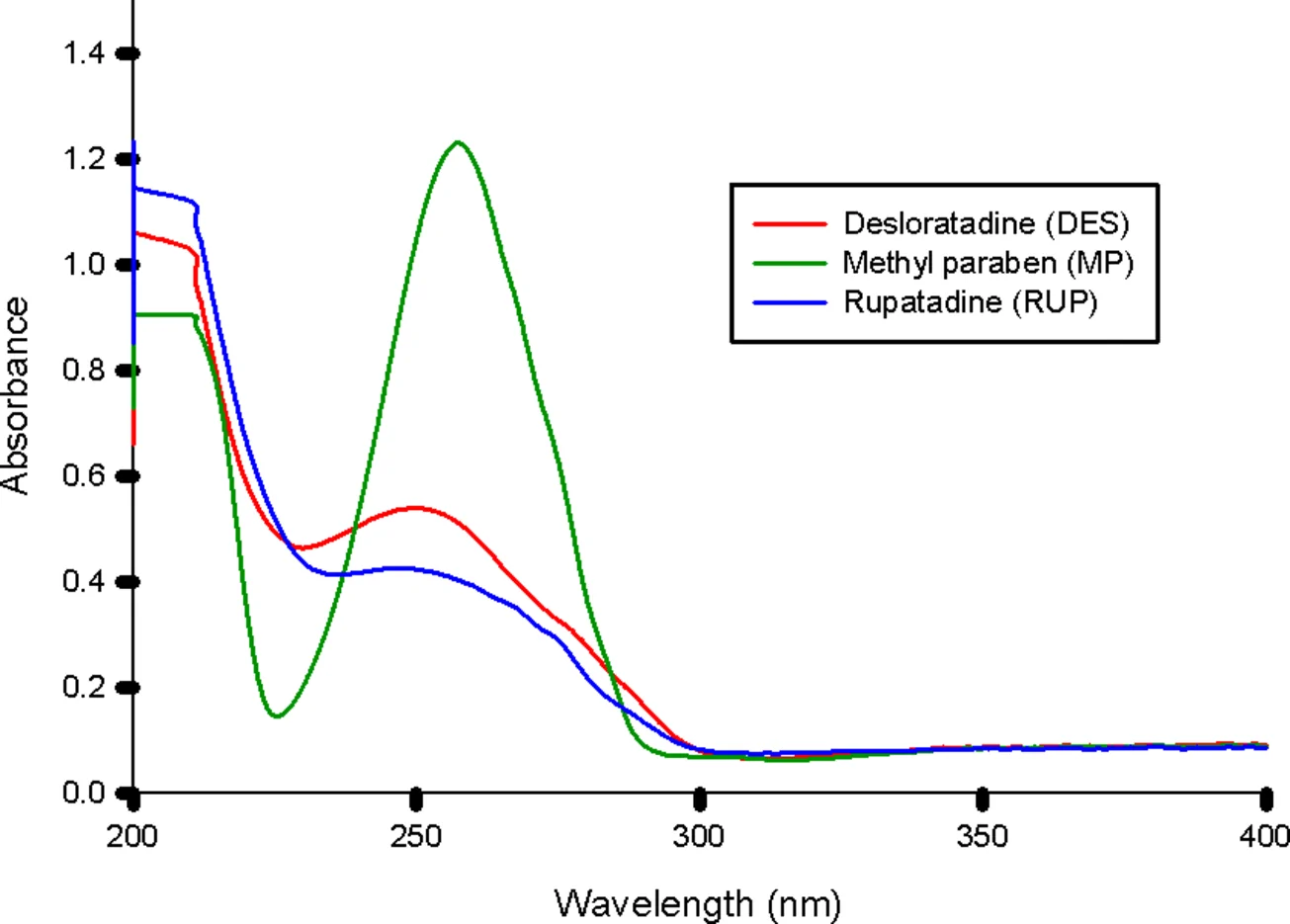
Zero-order absorption spectra of 10 µg mL^− 1^ of RUP, DES, and MP using ethanol as a blank.

Achieving a balance between analytical performance and practical efficiency remains a challenge^46^. The UV spectra of RUP, DES, and MP exhibit extensive overlap, as illustrated in Figure 2, which hampers their determination. In this study, the binary mixture of RUP and MP was quantified using an AI-assisted univariate spectroscopic method, DW; However, the dual-wavelength method, while effective for the binary mixture of RUP and MP, could not resolve DES in the ternary mixture due to extensive spectral overlap. Thus, it was accomplished using fast, reproducible, and highly sensitive multivariate chemometric techniques, including PLS-1, ANN, and MCR-ALS.

The method’s overall eco-friendliness was further evaluated using green assessment metrics, alongside visualization through the Glance framework.

### Method development

#### Dual wavelength (DW)

The principle of the dual wavelength approach is that the absorbance difference between two selected wavelengths in the mixture spectrum is directly proportional to the concentration of the target analyte^47^. It is based on selecting two wavelengths at which the interfering component exhibits identical absorbance values, so its contribution is effectively cancelled when the absorbance difference is calculated. In contrast, the component of interest shows a significant, concentration-dependent difference in absorbance between the two wavelengths, enabling its selective and accurate quantification^48^. The challenge is the selection of the wavelength pair corresponding to the highest selectivity and sensitivity. Manual scanning of spectral data is time-consuming, prone to human error, and often limited by subjective judgment, especially when evaluating subtle absorbance differences across hundreds of wavelength combinations. This study demonstrates the potential of AI to accelerate and enhance the development of analytical methods in pharmaceutical research. AI enables rapid, exhaustive analysis of the entire spectral range, automatically calculating absorbance differences for all possible wavelength pairs and applying precise selection criteria. This ensures that optimal pairs, such as those with maximum sensitivity for the analyte and complete cancellation of interference, are identified with high accuracy and reproducibility. Moreover, AI can visualize trends, detect flat regions, and highlight ideal zones for dual-wavelength applications, streamlining method development and enhancing analytical reliability. ^39^. AI tools facilitate rapid data analysis and support innovative methodological development without the need for complex software. Microsoft Copilot showed efficacy in optimizing wavelength selection for spectrophotometric drug analysis. It facilitated the identification of the most analytically reliable wavelength pair by systematically assessing recovery and precision parameters, thereby streamlining the overall method development process^39^.

**Fig. 3:**
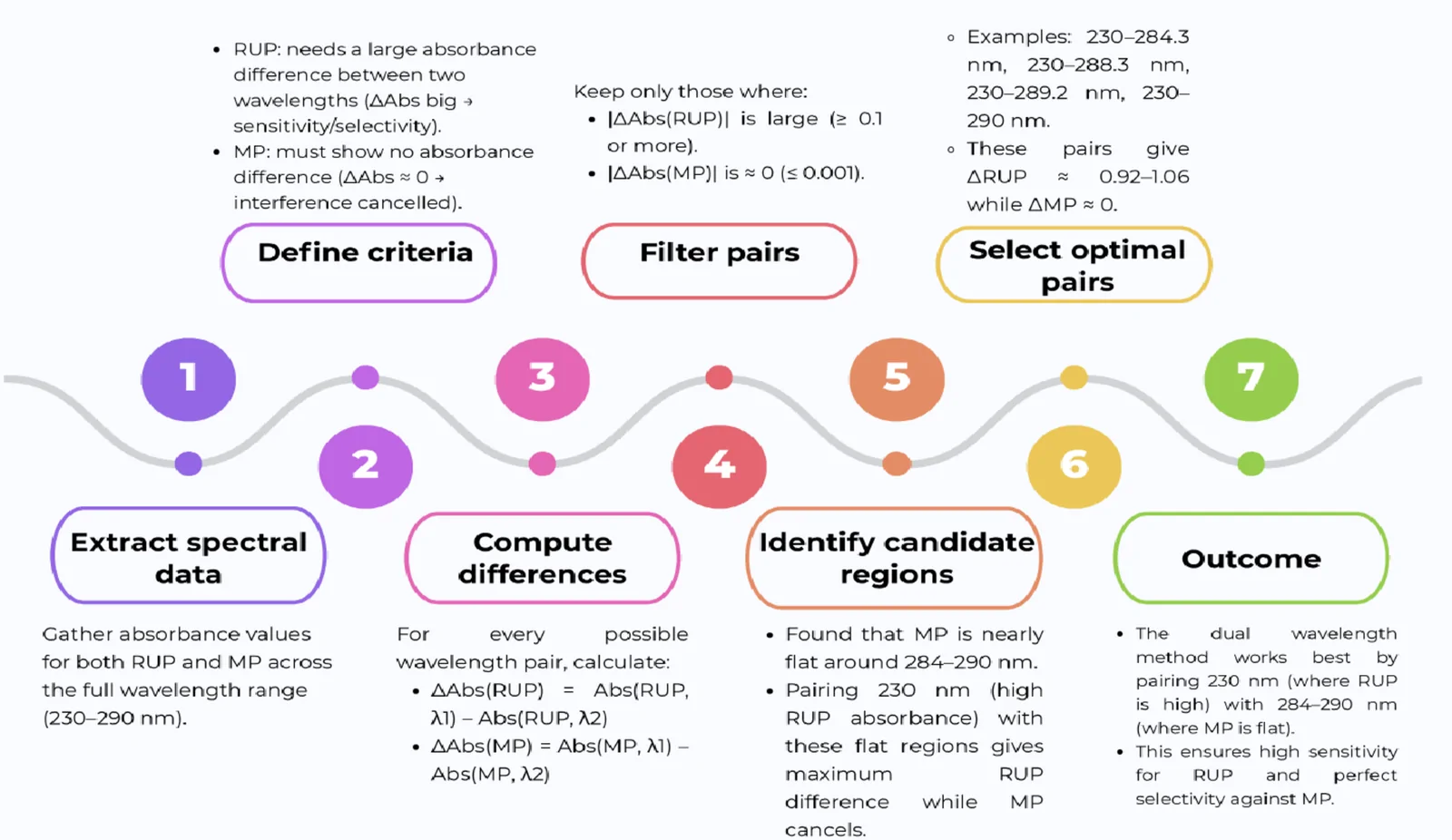
Flowchart illustrating the steps followed by the AI Copilot to determine the optimal wavelength pair.

For AI-assisted data analysis of the DW method, wavelength optimization was performed based on which pair would provide the highest sensitivity and selectivity. By only supplying Microsoft Copilot with both RUP and MP spectra, the final selection for spectra pairs was 230 and 284.3 nm for RUP using (ΔP = A₂₃₀ – A₂₈₄.₃), and 236.6 and 253.8 nm for MP using (ΔP = A₂₃₆.₆ – A₂₅₃.₈). To sum up, the process for selecting optimal wavelength pairs carried out by Microsoft Copilot started by defining criteria, ensuring that RUP shows a large absorbance difference for sensitivity, while the interfering component MP shows minimal difference. Next, spectral data are extracted across the full wavelength range (230–290 nm), and absorbance differences for all possible wavelength pairs are computed. Candidate regions are identified where MP is nearly flat, and RUP absorbance is high. From these, the optimal wavelength pairs are selected, maximizing sensitivity for RUP while cancelling interference from MP. The outcome ensures high selectivity and accurate quantification of RUP. Figures 3 illustrates the systematic steps followed by Microsoft Copilot to achieve the final selection of the optimal wavelength pair for RUP. The results presented in Table 2 demonstrate that the DW method is selective, validated, and suitable for the analysis of RUP and MP in various laboratory-prepared mixtures.

#### Multivariate methods

##### Calibration and validation sets

A calibration set was designed using a five-level, three-factor experimental design, along with an independent validation set. Spectral measurements of the prepared mixtures were recorded over the wavelength range of 200–400 nm. Careful selection of the spectral regions was essential to ensure the development of accurate and precise models. Wavelengths that did not provide informative absorbance signals were excluded to minimize noise. PLS-1, ANN, and MCR-ALS chemometric techniques were employed to achieve accurate quantification of RUP, DES, and MP without the need for any preliminary separation process. Regarding PLS-1, data points in the range of 227–277 nm were selected for RUP, while a range of 235–286 nm was chosen for DES. For MP, a narrower spectral region of 230–260 nm was chosen. These wavelength ranges were chosen because they contained the most informative spectral features of each analyte while excluding regions with negligible absorbance that could introduce unnecessary noise. The wavelength range 220–290 was selected for MCR-ALS, while 220–280 was used for ANN to retain sufficient spectral information for simultaneous resolution of the ternary mixture while minimizing uninformative spectral regions.

##### PLS-1

**Fig. 4:**
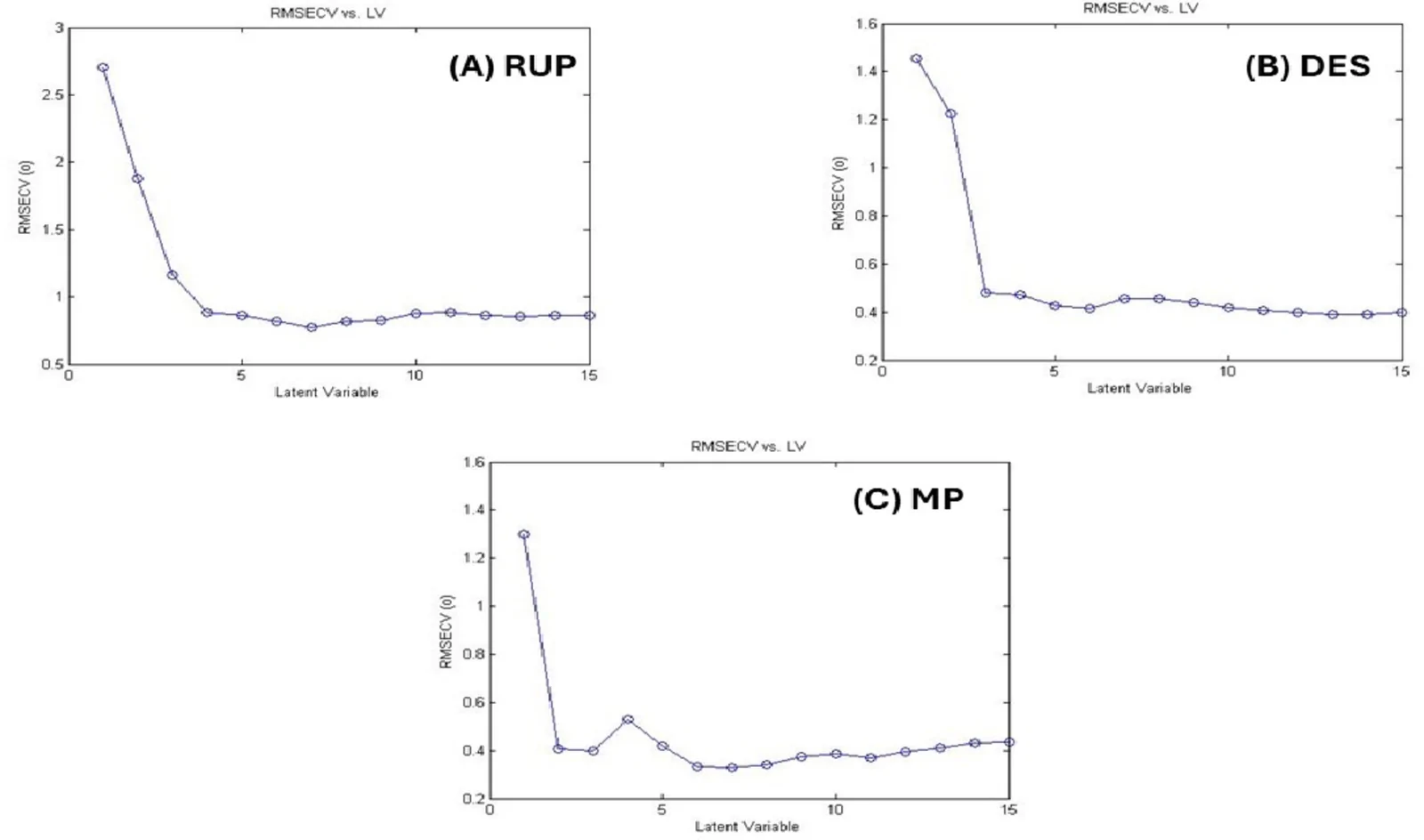
The optimum number of LVs for (A) RUP, (B) DES, and (C) MP.

This model is applied for factor analysis and is developed separately for each compound. It relies on a single concentration vector of the analyte of interest in the calibration set, which represents the principal difference between PLS-1 and PLS-2 models^15^. Accordingly, PLS-1 allows individual model optimization for each analyte, enabling tailored calibration performance instead of constructing a single global model for all three compounds simultaneously. During model construction, the appropriate number of latent variables (LVs) is selected separately for each analyte. LVs are mathematical factors extracted from the spectral data. They summarize the useful information related to analyte concentration while reducing noise and collinearity. This strategy is superior to adopting a unified number of LVs for all components, as the latter may negatively affect model robustness and quantitative performance. Therefore, precise determination of the optimal number of LVs is crucial to prevent overfitting and ensure reliable concentration predictions^49^. The optimal number of LVs was determined by minimizing the root mean square error of cross-validation (RMSECV) using the leave-one-out cross-validation method. In this procedure, each calibration sample was omitted once, predicted using a model built from the remaining samples, and the prediction errors were combined to calculate the RMSECV. The number of LVs corresponding to the lowest RMSECV was selected, as it provided the best predictive performance while minimizing the risk of overfitting. In the present study, 7, 6,7 LVs were found to be optimum for RUP, DES, and MP, respectively, yielding the lowest RMSECV values, as illustrated in Figure 4. Before model construction, the data were preprocessed by mean centering.

##### ANN

Artificial neural networks (ANNs) function through interconnected neuron-like units arranged in three main layers: input, hidden, and output layers^36^. In this study, a feedforward network architecture was implemented, and the learning process was carried out using the backpropagation algorithm^50,51^. The network configuration was systematically optimized to enhance predictive capability by varying key parameters, including the combinations of transfer functions, the training algorithm, and the number of neurons in the hidden layer. A trial-and-error strategy was adopted to identify the most suitable architecture. Three transfer function combinations, purelin–purelin, logsig–purelin, and tansig–purelin—were examined. Among them, the purelin–purelin configuration yielded superior predictive performance for all analytes. This outcome aligns with the inherently linear relationship between absorbance and concentration in the mixtures studied, favoring the use of linear activation functions. The performance and regressions showing MSE and R are presented in **Figures ****S1** and **S2.** The strong predictive performance of the model is confirmed by correlation coefficients (R) approaching unity for all datasets^52^. The optimized ANN architectures are summarized in **Table (S1)**.

##### MCR-ALS

**Fig. 5:**
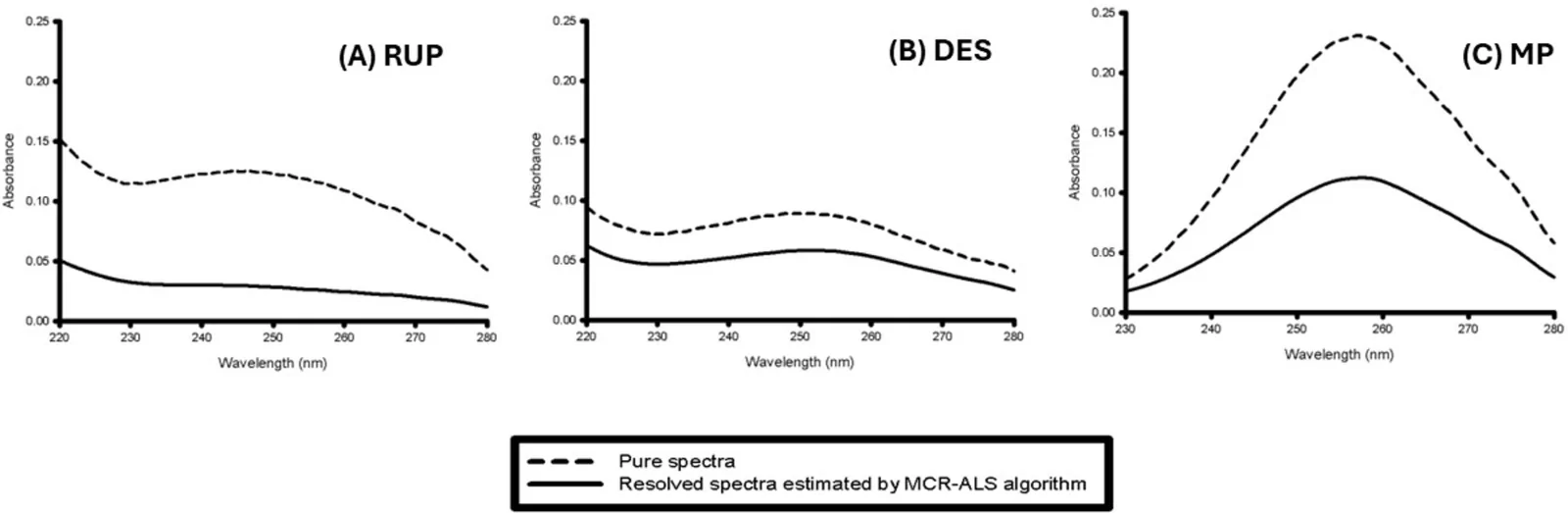
Pure spectra and resolved spectra estimated by the MCR-ALS algorithm for (A) RUP, (B) DES, and (C) MP.

MCR aims to recover the pure response profiles of unresolved mixture components without prior information by decomposing the data matrix according to a bilinear model. The method relies on ALS optimization under suitable constraints, yielding resolved spectral profiles, concentration profiles, and residual errors^53^. The initial estimates were obtained using evolving factor analysis (EFA) with a selected log eigenvalue threshold of − 2, resulting in a three-component model. Non-negativity constraints were imposed on both concentration and spectral profiles, along with correlation constraints applied to the concentration profiles^54^. The convergence criterion was set to 0.1% and was achieved after 6 ALS iterations. The model exhibited an acceptable fit, with a lack-of-fit value of 5.0017% and a variance explained (R²) of 99.8%. Together, these values indicate that the MCR-ALS model provides a good mathematical description of the spectral data. The resolved pure spectral profiles, shown in Figure 5, closely resemble the experimentally measured pure spectrum, confirming the validity of the MCR model.

##### Application of the proposed models on the validation set

To validate the predictive performance of the proposed chemometric methods, they were applied to an independent validation set consisting of six mixtures. To further evaluate model robustness in the presence of trace levels of degradation products and/or preservatives, three mixtures in the validation set were deliberately prepared with outlier concentrations of the degradation products and the preservative. This scenario commonly reflects practical pharmaceutical analysis of pure drug substances and finished dosage forms. All proposed multivariate models successfully quantified RUP, DES, and MP as summarized in Table 3, demonstrating their suitability for impurity profiling and quality control applications. As shown in Table 3, ANN provided the most accurate and precise predictions for all three analytes, with RSD values below 1.5%, compared to PLS-1 (RSD < 2.1%) and MCR-ALS (RSD up to 3.9%). This confirms the superiority of ANN for resolving complex ternary mixtures, particularly in the presence of the preservative MP.

### Regression and validation parameters for the proposed univariate and multivariate methods

Table 4 summarizes the regression and validation parameters of the proposed spectrophotometric methods for the determination of RUP, DES, and MP, with validation performed in accordance with ICH guidelines^55^. The methods were validated by evaluating linearity, selectivity, precision, and accuracy. Additionally, the root mean square error of calibration (RMSEC) and prediction (RMSEP) for each component was calculated for the proposed multivariate models. The lower RMSEC and RMSEP values for ANN compared to PLS-1 and MCR-ALS across all analytes further demonstrate its enhanced predictive capability.

### Application of proposed univariate and multivariate methods on pharmaceutical dosage form

When the proposed methods were applied to the determination of RUP in Alergoliber^®^ syrup, satisfactory recovery values were obtained **Table (S2)**. The performance of the proposed chemometric methods was statistically compared with that of the reported method^16^ using Student’s *t*-test and the variance ratio *F*-test. The results showed no significant differences between the methods, confirming the good predictive capability of the proposed models, as presented in **Table (S2).**

### Comparative study of univariate and multivariate methods

The analytical performance of the AI-assisted univariate DW method and the three multivariate chemometric techniques (PLS-1, ANN, and MCR-ALS) was systematically compared to evaluate their respective strengths and limitations for the simultaneous determination of RUP, DES, and MP.

#### Univariate method (DW)

The DW method, guided by AI for optimal wavelength selection, demonstrated excellent linearity, precision, and sensitivity for the binary mixture of RUP and MP, with mean recoveries of 101.22% and 101.29% and %RSD values below 1.0% (Table 2). However, its application was fundamentally limited to binary mixtures, as it could not resolve DES in the ternary mixture due to the extensive spectral overlap among all three components. This limitation highlights the inherent constraint of univariate methods when faced with complex multi-component systems, particularly those containing a preservative (MP) alongside an active pharmaceutical ingredient and its impurity.

**Table 2:** Results obtained for the determination of RUP and MP in laboratory-prepared mixtures by the DW method.

Conc. of lab. mix. µg/mL		*R*%	
RUP	MP	RUP	MP
40	4	101.27	101.94
40	6	100.80	100.12
40	8	101.58	101.82
**Mean**		101.22	101.29
**SD**		0.39	1.02
**%RSD**		0.39	1.00

#### Multivariate methods

In contrast, all three multivariate chemometric techniques successfully resolved the ternary mixture, enabling simultaneous quantification of RUP, DES, and MP without any preliminary separation. The predictive performance of each method was evaluated using the independent validation set (Table 3), with the following key observations:

**Table 3:** Percentage recoveries of RUP, DES, and MP in the validation set using the proposed models.

Mixture no.	RUP	Calibration models %Recovery ^a^		
		PLS-1	ANN	MCR-ALS
**26**	2	98.30	103.00	94.97
**27**	3	103.84	101.33	99.19
**28**	6	103.26	99.67	104.92
**29**	5	99.94	100.00	99.43
**30**	6	100.57	99.67	98.32
**31**	8	102.21	99.25	104.37
**Mean**		101.35	100.49	100.20
**RSD%**		2.09	1.41	3.79
**Mixture no.**	**DES**	**Calibration models** **%Recovery** ^**a**^		
		**PLS-1**	**ANN**	**MCR-ALS**
**29**	2	102.90	103.00	106.00
**30**	3	99.20	101.33	98.08
**31**	4	100.43	100.50	101.51
**Mean**		100.85	101.61	101.86
**RSD%**		1.86	1.25	3.91
**Mixture no.**	**MP**	**Calibration models** **%Recovery** ^**a**^		
		**PLS-1**	**ANN**	**MCR-ALS**
**29**	5	99.33	100.00	97.92
**30**	6	102.25	99.67	101.74
**31**	8	98.68	99.25	99.43
**Mean**		100.09	99.64	99.70
**RSD%**		1.90	0.38	1.93

^**a**^ average of three determinations.

**Fig. 6:**
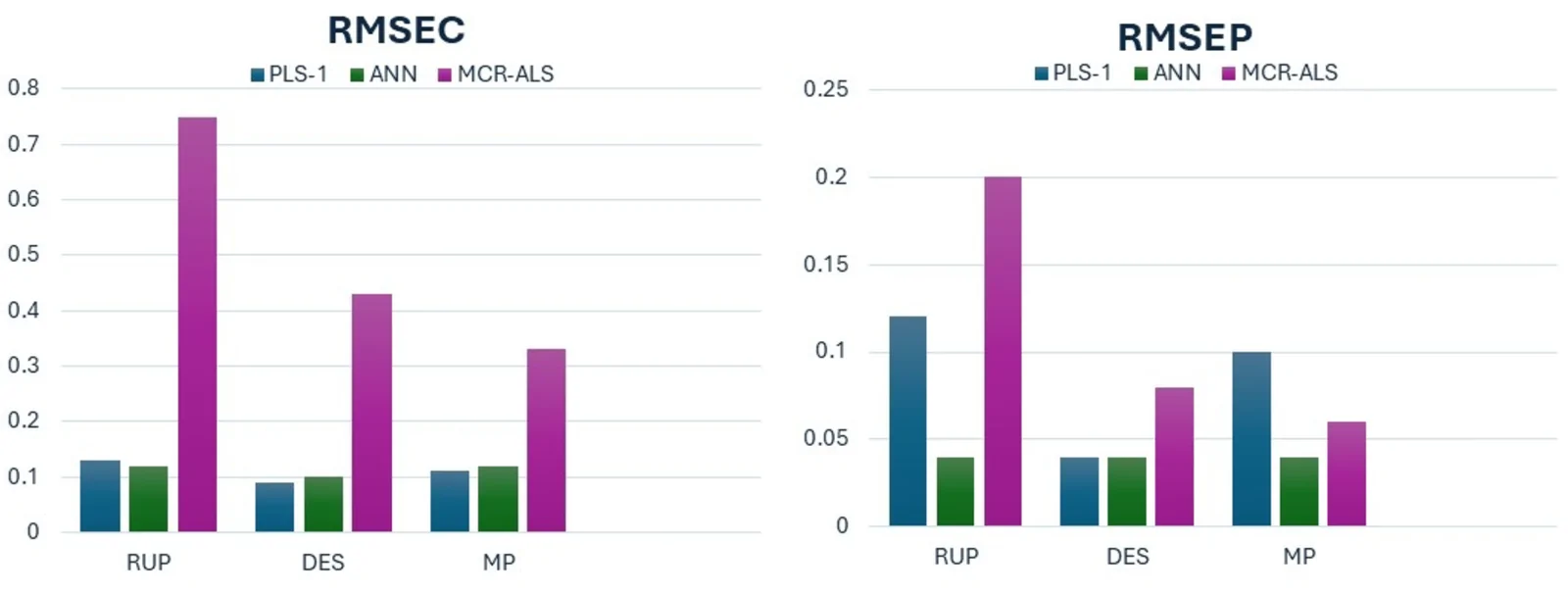
Comparison between RMSEC and RMSEP of the three components using the proposed multivariate methods.

ANN demonstrated the best overall performance, yielding mean recoveries closest to 100% for all three analytes (RUP: 100.49%, DES: 101.61%, MP: 99.64%) with the lowest RSD values (1.41%, 1.25%, and 0.38%, respectively). The RMSEP values for ANN were the lowest among all methods (RUP: 0.04, DES: 0.04, MP: 0.04), confirming its superior predictive accuracy **(**Table 4; Fig. 6**)**. PLS-1 showed comparable accuracy with mean recoveries of 101.35% (RUP), 100.85% (DES), and 100.09% (MP), and slightly higher RSD values (2.09%, 1.86%, and 1.90%). Its RMSEP values (RUP: 0.12, DES: 0.04, MP: 0.10) were higher than ANN for RUP and MP, indicating slightly lower precision for these analytes. MCR-ALS successfully resolved the mixture with mean recoveries of 100.20% (RUP), 101.86% (DES), and 99.70% (MP), but exhibited the highest RSD values (up to 3.91%) and RMSEP values (RUP: 0.20, DES: 0.08, MP: 0.06), indicating comparatively lower precision.

**Table 4:** Regression and validation parameters of the established univariate and multivariate methods for the determination of RUP, DES, and MP.

Validation parameters	RUP				DES				MP			
	D^0^	PLS-1	ANN	MCR-ALS	D^0^	PLS-1	ANN	MCR-ALS	D0	PLS-1	ANN	MCR-ALS
**Linearity range (µg/mL)**	4–40	2–10			NA	1–5			3–20	2–10		
**Correlation coefficient (r)**	0.999	0.9992	0.9991	0.9638	NA	0.9978	0.9978	0.9573	0.9996	0.9992	0.9991	0.9933
**Slope**	0.0238	0.9969	1.0024	1	NA	0.9827	0.9783	1	0.0692	0.9957	0.9997	1
**Intercept**	− 0.0355	0.0977	-0.0494	-6.02 × 10^− 16^	NA	0.063	0.0984	-3.12 × 10^− 16^	0.0283	0.0129	0.0177	-8.33 × 10^− 16^
**RMSEC** ^**a**^	-	0.13	0.12	0.75	NA	0.09	0.10	0.43	-	0.11	0.12	0.33
**RMSEP** ^**b**^	-	0.12	0.04	0.20	NA	0.04	0.04	0.08	-	0.10	0.04	0.06
**Accuracy** ^**c**^ **(Mean% ± SD)**	100.55 ± 1.20	101.35 ± 2.12	100.49 ± 1.42	100.20 ± 3.80	NA	100.85 ± 1.88	101.61 ± 1.27	101.86 ± 3.98	100.65 ± 1.34	100.09 ± 1.90	99.64 ± 0.38	99.70 ± 1.93
**Precision** ^**d**^ **(%RSD)**												
**Repeatability**	0.63	-	-	-	NA	-	-	-	0.06	-	-	-
**Intermediate**	1.63	2.09	1.41	3.79	NA	1.86	1.25	3.91	0.05	1.90	0.38	1.93
**LOD** ^**e**^**(µg/mL)**	0.57	-	-	-	NA	-	-	-	0.48	-	-	-
**LOQ** ^**e**^ **(µg/mL)**	1.71	-	-	-	NA	-	-	-	1.46	-	-	-

Shaded cells: data of the straight line plotted between predicted concentrations of each component versus actual concentrations. ^**a**^ Root Mean Square Error of Calibration. ^**b**^ Root Mean Square Error of prediction. ^c^ Average of three different concentrations repeated three times within the day (D^0^) and average of the concentrations in the validation set mixtures (PLS-1, ANN, and MCR-ALS.). ^d^ Precision: %RSD of three concentrations measured on the same day (repeatability), and on three different days (intermediate) for D^0^ and the %RSD of the concentrations in the validation set mixtures measured in three days (intermediate) for PLS-1, ANN, and MCR-ALS. ^e^ LOD and LOQ were calculated from the standard deviation of the residual (s) and the slope of the calibration curve (S) according to the following equations: LOD = 3.3(s/S) and LOQ = 10(s/.

The results clearly demonstrate that multivariate methods are essential for resolving the complex ternary mixture containing RUP, DES, and MP, where the univariate DW method failed. Among the multivariate approaches, ANN emerged as the superior technique, consistently providing the most accurate and precise predictions for all three analytes. This superiority can be attributed to ANN’s ability to model non-linear relationships and complex spectral interactions that may arise in multi-component systems, particularly in the presence of the preservative MP which exhibits significant spectral overlap with both RUP and DES (Fig. 2). PLS-1, while slightly less accurate than ANN, remains a robust and interpretable alternative suitable for routine quality control applications. MCR-ALS, despite its higher prediction errors, offers the advantage of recovering pure spectral profiles (Fig. 5), providing valuable qualitative insights into the mixture composition. Overall, the performance ranking was ANN > PLS-1 > MCR-ALS, confirming the superiority of machine learning approaches for complex multi-component pharmaceutical analysis, while the AI-assisted DW method remains a simple and effective option for binary mixtures.

**Fig. 7:**
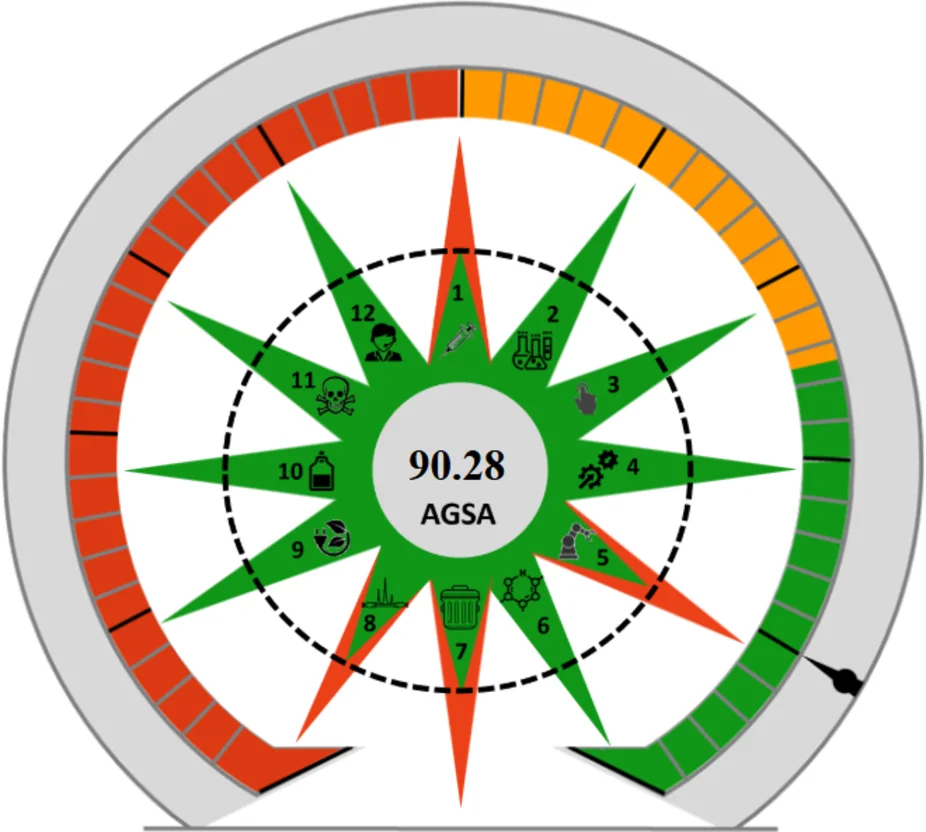
AGSA for the developed spectrophotometric method.

### Green analytical chemistry assessment

GAC emphasizes environmentally responsible analytical practices utilizing safer solvents, waste reduction, energy efficiency, and simplified sample preparation. In this study, several methodological choices were intentionally aligned with the twelve principles of GAC, and the environmental performance of the developed method was quantitatively evaluated using the new AGSA tool. Ethanol was selected as the primary solvent owing to its renewable origin, low toxicity, and biodegradability, thereby supporting the principles of renewable reagents and reduced hazard. The integration of AI-assisted experimental design with chemometric techniques represents a transformative approach in modern analytical chemistry, particularly in promoting sustainability and efficiency. Together, these strategies not only streamline method development but also align with the principles of GAC by minimizing chemical waste, lowering energy requirements, and shortening experimental timelines. The AGSA assessment indicated moderate sample preparation limited to dilution, no derivatization steps, and the use of a low-energy UV spectrophotometer. The procedure employed semi-miniaturized conditions and generated less than 100 mL of waste per sample, which was safely managed. High operator safety was maintained with minimal personal protective equipment. Overall, the method achieved an AGSA score of 90.28%, demonstrating good compliance with GAC principles, Figure 7.

### The spectrophotometric methods visualization using the GLANCE framework

To enhance clarity, we applied the recently introduced GLANCE framework^40^. GLANCE visualization is an analytical tool that provides a clear overview of complex datasets, enabling rapid interpretation of multidimensional information. Combining graphical representation with statistical insights, it allows researchers to identify patterns, trends, and correlations efficiently. Through color-coded maps, clustering, and interactive elements, GLANCE highlights outliers and key variables, supporting informed decision-making and optimization of experimental conditions. By transforming complex data into an accessible visual format, it enhances both the speed and clarity of data interpretation in data-driven research^40^.

**Fig. 8:**
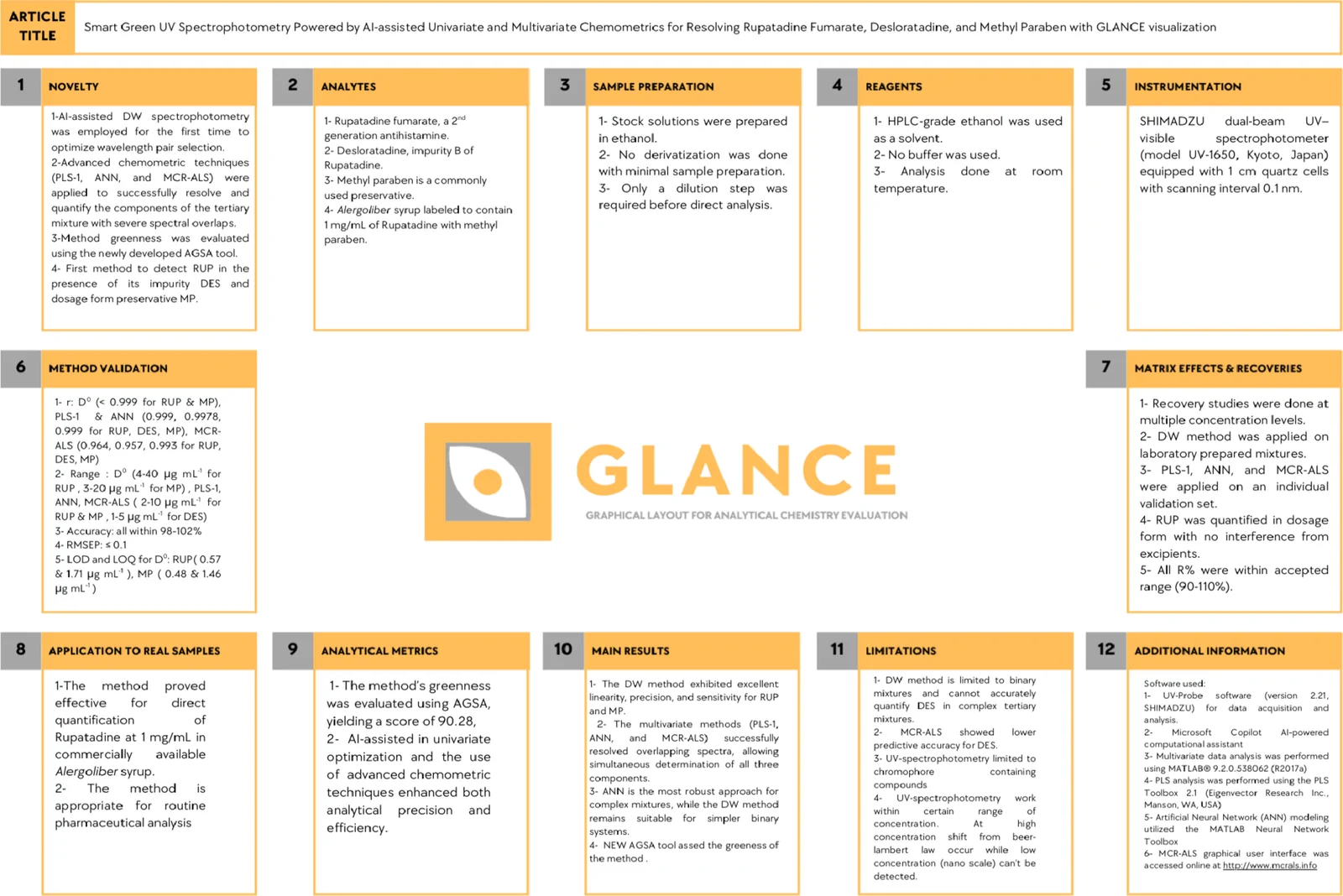
GLANCE visualization of the proposed spectrophotometric method for the analysis of RUP, DES, and MP.

The GLANCE framework (Fig. 8) provides a holistic visual summary of all method attributes, enabling rapid assessment and comparison of the AI-assisted univariate and multivariate chemometric approaches.

## Conclusion

In conclusion, the proposed green spectrophotometric techniques offered a simple, rapid, and reliable approach for the simultaneous determination of RUP, its hepatotoxic impurity DES, and the preservative MP. The AI-assisted dual-wavelength method proved adequate for the binary mixture of RUP and MP, demonstrating the power of artificial intelligence in streamlining univariate method development. However, for the more complex ternary mixture including DES, univariate methods were insufficient, necessitating the use of multivariate chemometrics.

Among the multivariate approaches, ANN demonstrated better predictive performance (lowest RMSEP, highest precision), followed by PLS-1 and MCR-ALS. This ranking confirms the superiority of machine learning techniques for resolving highly overlapped spectra in multi-component pharmaceutical systems, particularly in the presence of preservatives that exhibit complex spectral interactions. PLS-1 remains a robust and interpretable alternative for routine quality control, while MCR-ALS offers valuable qualitative insights through spectral recovery.

All methods were successfully applied to pharmaceutical dosage forms and validated according to ICH guidelines, with the multivariate methods enabling comprehensive impurity and preservative profiling. The methods achieved an impressive AGSA greenness score of 90.28%, confirming their alignment with sustainable analytical chemistry principles. Furthermore, the GLANCE framework provided a comprehensive visual summary of all method attributes, enhancing clarity and facilitating method comparison.

This integrated approach combining AI-assisted univariate method development with comparative multivariate chemometrics and comprehensive greenness assessment—establishes a new paradigm for environmentally conscious pharmaceutical analysis of complex mixtures containing active ingredients, toxic impurities, and preservatives.

## Supplementary Information

Below is the link to the electronic supplementary material.

Supplementary Material 1

## Data Availability

The data are available from the corresponding author on reasonable request.
