# Supplementary material for: “Green spectrophotometric determination of rupatadine, its toxic impurity, and methyl paraben: comparative AI-assisted univariate versus multivariate chemometrics”
Source: Sci Rep. 2026 Sep 9;16:28212. doi: 10.1038/s41598-026-68686-1 (PMC13558692; doi:10.1038/s41598-026-68686-1)
Supplement: Supplementary file 1 [file 41598_2026_68686_MOESM1_ESM.docx]

**Supplementary data**

**Figure (S1): Best validation performance for the prediction of artificial neural networks model**

**Figure (S2): Prediction for the training, test and validation diagrams of ANN model**

**Table (S1):** **Optimized parameters of ANNs**

| **Method** | **ANN** | | |
| --- | --- | --- | --- |
| **Constituent** | RUP | DES | MP |
| **Hidden neurons number** | 5 | 5 | 5 |
| **Transfer functions** | purelin-purelin | | |
| **Learning coefficient** | 0.001 | | |
| **Learning coefficient decrease** | 0.1 | | |
| **Learning coefficient increase** | 10 | | |

**Table (S2): Statistical comparison of the results obtained by the proposed methods and the reported method for the analysis of RUP in Alergoliber^®^ Syrup**

| **Parameter** | **D^o^** | **PLS-1** | **ANN** | **MCR-ALS** | **Reported method^c^ [1]** |
| --- | --- | --- | --- | --- | --- |
| **Mean ^a^** | 100.97 | 101.73 | 101.33 | 101.97 | 102.95 |
| **SD** | 2.4 | 2.10 | 2.08 | 2.19 | 1.29 |
| **N** | 9 | 9 | 9 | 9 | 6 |
| **Variance** | 5.76 | 4.41 | 4.33 | 4.80 | 1.66 |
| **Student's t-test (2.160)^b^** | 2.06 | 1.39 | 1.86 | 1.09 | - |
| **F value (4.8183)^b^** | 3.47 | 2.66 | 2.61 | 2.90 | - |

**^a^** Average of three determinations.

**^b^** Values between parenthesis correspond to the theoretical values of t and F (p =0.05).

^c^ HPLC nethod with Micellar mobile phase: 0.13 M SDS, 0.1 M Na₂HPO₄ (pH 2.8) with 10% n-butanol; 45 °C; detection at 267 nm.

[1] M.M. Amer, A.A. Habib, S.F. Hammad, A.H. Kamal, Green micellar stability‐indicating high‐performance liquid chromatography method for determination of rupatadine fumarate in the presence of its main impurity desloratadine: Oxidative degradation kinetics study, Journal of Separation Science, 46 (2023) 2300135.
